# Soil-transmitted helminth (STH) infections in the Wolaita zone in Southern Ethiopia: mid-stage evaluation of the Geshiyaro project and progress towards the interruption of transmission

**DOI:** 10.1186/s13071-024-06422-2

**Published:** 2024-08-21

**Authors:** Birhan Mengistu, Ewnetu Firdawek Liyew, Melkie Chernet, Geremew Tasew, Rosie Maddren, Benjamin Collyer, Ufaysa Anjulo, Adugna Tamiru, Kathryn Forbes, Zelalem Mehari, Kebede Deribe, Teshale Yadeta, Mihretab Salasibew, Getachew Tollera, Roy Anderson

**Affiliations:** 1 London Centre for Neglected Tropical Disease Research, Department of Infectious Disease Epidemiology, Faculty of Medicine, St Marys Campus, Imperial College London, London, UK; 2 Bacterial, Parasitic and Zoonotic Disease Research Directorate, Ethiopian Public Health Institute, Addis Ababa, Ethiopia; 3 Disease Prevention and Health Promotion Core Process, Ministry of Health, Addis Ababa, Ethiopia; 4 Children’s Investment Fund Foundation, London, UK

**Keywords:** Soil-transmitted helminths (STH), Prevalence, Intensity, Mass drug administration (MDA)

## Abstract

**Background:**

This paper documents changes in the prevalence and intensity of soil-transmitted helminth (STH) infections in the Geshiyaro project in the Wolaita zone of Southern Ethiopia.

**Methods:**

The Geshiyaro project comprises three intervention arms. Arm 1 is subdivided into the Arm 1 pilot (one district) and Arm 1 (four other districts), both receiving integrated community-wide mass drug administration MDA (cMDA) with intensive water, sanitation, and hygiene (WaSH) interventions. Arm 2 involves 18 districts with cMDA interventions plus the existing government-led One WaSH program, while Arm 3 serves as a control with school-based MDA (sMDA) interventions plus the existing government-led One WaSH program in three districts. The study is designed as a cohort investigation over time, with the establishment of longitudinal sentinel sites where infection levels are assessed annually. A total of 45 longitudinal parasitological surveillance sentinel sites are being used across all three intervention arms to monitor STH prevalence and intensity of infection. From each of the 45 sentinel sites, 150 individuals were randomly selected, stratified by age and gender. The *t*-test and analysis of variance (ANOVA) were employed to compare infection prevalence and intensity across the three study arms over time.

**Results:**

The prevalence of STH decreased significantly from 34.5% (30.6%, 38.5%) in 2019 to 10.6% (8.3%, 13.4%) in 2022/2023 (*df* = 1, *P* < 0.0001) in the Arm 1 pilot, from 27.4% (25.2%, 29.7%) in 2020 to 5.5% (4.4%, 6.7%) in 2023 (*df* = 1, *P* < 0.0001) in Arm 1, from 23% (21.3%, 24.8%) in 2020 to 4.5% (3.7%, 5.3%) in 2023 (*df* = 1, *P* < 0.001) in Arm 2, and from 49.6% (47.4%, 51.7%) in 2021 to 26.1% in 2023 (*df* = 1, *P* < 0.0001) in Arm 3. The relative reduction in the prevalence of any STH was the highest in the arms employing cMDA, namely Arm 2, with a decrease of 82.5% (79.3%, 84.2%), followed by Arm 1 with a reduction of 80.1% (75.3%, 84.6%), and then the Arm 1 pilot with a decrease of 69.4% (60.1%. 76.6%). Arm 3 employing sMDA had the lowest decrease, with a reduction of 46.9% (43.6%, 51%). The mean intensity of infection (based on Kato–Katz egg count measures) for *Ascaris lumbricoides* species, which was the dominant STH species present in the study area, decreased significantly in Arms 1 and 2, but only slightly in Arm 3. The prevalence of hookworm and *Trichuris trichiura* infections were found to be very low in all arms but also decreased significantly.

**Conclusions:**

The reduction in the prevalence and intensity of STH in Arms 1 and 2 revealed steady progress towards transmission interruption based on cMDA intervention, but additional efforts with MDA coverage and WaSH interventions are needed to achieve a prevalence threshold < 2% based on the quantitative polymerase chain reaction (qPCR) diagnostic method.

**Graphical Abstract:**

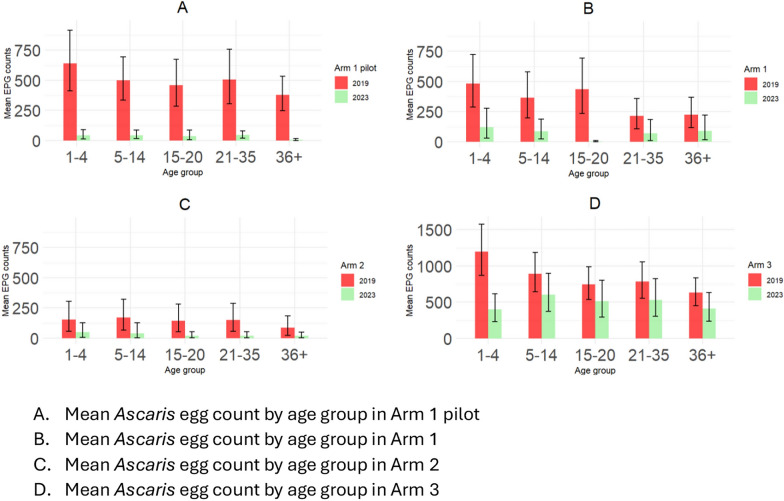

**Supplementary Information:**

The online version contains supplementary material available at 10.1186/s13071-024-06422-2.

## Background

Soil-transmitted helminth (STH) infection is classified as a neglected tropical disease (NTD) by the World Health Organization (WHO). These infections are very common in tropical regions across the world, particularly affecting the world’s poorest populations. WHO estimates that more than 1 billion individuals are infected with STHs each year. The disease is predominantly found in sub-Saharan countries, Asia, and Latin America, where there is poor sanitation and limited access to safe water supply, which results in a wide range of health, social, and economic problems [1]. The control and elimination of the morbidity caused by STH infection focuses on the distribution of albendazole (ALB) or mebendazole through mass drug administration (MDA) to school-age children (SAC), preschool-age children (pre-SAC), adolescent girls, women of reproductive age (WRA), and adults working in high-risk occupations. However, due to the shortage in the supply of donated drugs, WHO recommends that priority be given to SAC. Repeated treatments are required, since helminth infections do not elicit strong acquired immunity following primary infection. The frequency of treatment is adjusted based on the population’s prevailing prevalence of infection, which is viewed as a marker of transmission intensity in a defined setting [2, 3].

STH infections are widely prevalent in Ethiopia. The first nationwide mapping survey, which was conducted from 2013 to 2015, revealed that 96.7 million people lived in districts exposed to STH infections caused by *Ascaris lumbricoides*, hookworm, and *Trichuris trichiura* [4]. The national prevalence of STH infections was estimated at 21.7% (*A. lumbricoides*: 12.8%, hookworm: 7.6%, *T. trichiura*: 5.9%) [5]. The survey indicated that the highest prevalence of STH was in the Southern Nations, Nationalities, and Peoples’ Region (SNNPR) of Ethiopia (42.4%), particularly in the Wolaita zone where the Geshiyaro project is being implemented [5].

A national helminth deworming program was launched in Ethiopia in 2015 with the aim of eliminating STH as a public health problem. All STH endemic districts received sMDA according to WHO guidelines [6]. Despite this treatment continuing until 2017, the decrease in STH infection rates was not significant [6]. The factors contributing to the limited impact on STH prevalence include poor uptake of MDA, low coverage of clean water, lack of improved sanitation, and limited behavioral interventions to inform communities about STH infection control [4].

Surveys conducted after the sMDA introduction in different countries have revealed a reduction in STH prevalence after 4 years of treatment in SAC, with a lesser degree of decrease seen in adults [7]. In Congo, a 3-year school-based MDA (sMDA) treatment program reduced the prevalence of STH from 97% to 67% [8]. Similarly, in Zanzibar, 3 years of sMDA reduced *A. lumbricoides* prevalence from 36% to 22% [9]. However, maintaining a low to moderate level of prevalence after cessation of treatment has proved to be difficult in many endemic areas, especially in the absence of improvements in water, sanitation, and hygiene (WaSH) infrastructure and with MDA only focused on SAC. In addition, repeated annual rounds of MDA administration over many years are challenging in regions with scarce health resources and many other disease treatment priorities, with reduced compliance to treatment among individuals exposed to many rounds of MDA. Hence, the expansion of MDA to the entire community combined with improved WaSH and behavioral change communication (BCC) has been suggested to facilitate progress towards the elimination or interruption of STH transmission [8–10].

The Geshiyaro project, meaning “clean inside and out” in the local Wolaita language, is designed to assess the feasibility of interrupting the transmission of STH through high coverage of cMDA (> 90% coverage target) and improvements in WaSH infrastructure implemented within the existing government-led NTD control program. It is not a clinical trial but a proof-of-concept study in order to demonstrate that expanding MDA community-wide and increasing coverage to levels of 90%-plus and improving WaSH can drive progress towards STH transmission interruption while implementing the intervention within the framework of the Ministry of Health program for NTD control. The project started in 2019 but the planned full scale-up of the implementation was severely impacted by the COVID-19 pandemic in 2020 and 2021, which delayed initiation of all the arms and failure to deliver all the planned rounds of MDA [11].

The Geshiyaro study encompasses three arms involving various evaluation methods to help assess the impacts of the different components. Arm 1 and Arm 2 districts are in the Wolaita zone, Southern Ethiopia, and serve as intervention groups, whereas Arm 3 is a control group, located outside the Wolaita zone, in which the current sMDA programs continue without enhancement. Arm 3 districts, found in the Oromia and Sidama regions, were selected based on their similarity in socioeconomic characteristics to the intervention arms. Interventions in Arm 1 include expanded cMDA, improved WaSH infrastructure targeting the provision of both water and basic sanitation coverage to over 80% of households, and the introduction of BCC programs in all the communities within this arm. In Arm 2, there is cMDA and the existing government-led ONE WASH NATIONAL PROGRAM (OWNP). In Arm 3, the national sMDA is implemented alongside OWNP. The OWNP program in Ethiopia is an approach that aims to enhance water and sanitation provision throughout the country. While this program has made progress in improving WaSH sectors in small towns, it faces challenges in many rural areas due to limited funding and coordination issues among stakeholders, resulting in slower overall improvements [12].

The objective of the Geshiyaro project is to assess the feasibility of interrupting transmission of both STH and schistosome infections on a large scale within an NTD control program run by the Federal Ministry of Health (FMoH) in Ethiopia. This paper focuses on STH infection and presents interim findings over the first 5 years of the project’s duration, analyzing the change in prevalence and intensity of infection using parasitological data gathered from longitudinal sentinel survey sites. The project protocol is detailed in an earlier publication [13]. In addition, baseline prevalence and intensity of infection data and preliminary WaSH findings have been published previously [14, 15].

## Methods

### Study area

**Fig. 1:**
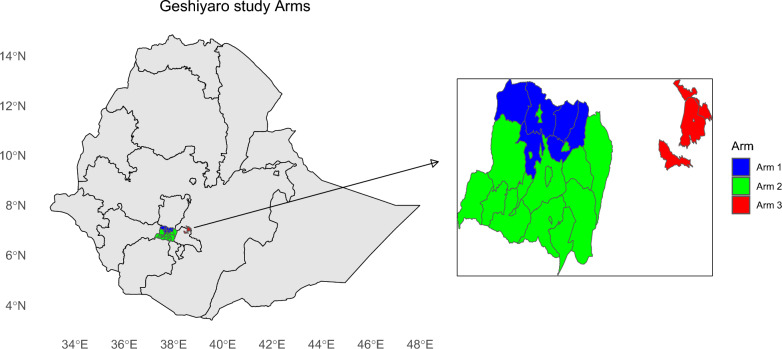
Map showing the districts in the three arms of the Geshiyaro project within Ethiopia

The study area is located in the Wolaita zone in the SNNPR (now Southern Ethiopia) of Ethiopia, home to an estimated 2.4 million people [16]. Sodo town is the zonal administrative center, which is located 330 km southwest of Addis Ababa. The Wolaita zone shares borders with the Sidama region, while Sidama shares borders with Oromia. There are 23 districts in the Wolaita zone (increased from 15 at the 2018 baseline due to the re-definition of district boundaries), grouped in either Arm 1 or Arm 2. The three control districts in Arm 3 are found in the nearby Sidama and Oromia regions (Fig. 1).

### Study design

The study is designed as a longitudinal cohort which involves the establishment of annual longitudinal sentinel sites [13]. These sites serve as cohorts to investigate longitudinal trends in the prevalence and intensity of infection reduction over time, explore the association between infection and treatment coverage and individual compliance to treatment, and examine the relationship between infection levels and access to WaSH facilities. The sample size was calculated to determine at least a 10% decrease from the initial prevalence. The participants were selected to represent all demographic groups proportionally. As a result, it involved a sample of 150 individuals per sentinel site, with a total of 45 sites (15 from districts in each arm) designated. The design involves monitoring a broader range of age classes and both genders, including adults and pre-SAC, in addition to SAC. The sentinel sites were selected after baseline parasitological mapping and stratification by co-endemicity of STH and schistosome infections, to ensure a balance in sites across different infection intensity categories (i.e., low, medium, and high). The participants in the sentinel sites were identified randomly from households based in the identified site, with consent obtained from study participants for their participation [13].

### Project implementation

The implementation of the Geshiyaro project was started in a pilot site in Bolosso Sore district of the Wolaita zone in 2018 to test intervention procedures and parasitological data collection methods. The cMDA, WaSH, and BCC interventions were scaled up to all other districts in Arms 1 and 2 by the end of 2020. The scale-up faced significant delays as a result of the COVID-19 pandemic, which required redirecting resources from NTD control activities to the control of the SARS-CoV-2 epidemic in Ethiopia. The project was initially intended to be completed by the end of 2022/2023 after 5 years of implementation, but has been extended until 2024/2025, with final evaluation in 2026, to address interventions missed or delayed due to the COVID-19 pandemic.

The administration of MDA is implemented by the FMoH through the health extension (HE) program, a major pillar of the health system in Ethiopia [17]. This program uses health extension workers (HEWs), who are government employees mandated to provide primary healthcare services in the community, including MDA. The HEWs visit house-to-house to treat eligible households for STH using ALB. In the Geshiyaro project, smartphones and scanners are used by HEWs to record fingerprint, demographic, and treatment data in five selected districts of the Geshiyaro project (three from Arm 1 and two from Arm 2), which are referred to as biometric districts. Paper-based records are employed to register MDA data in all other districts.

The smartphones are used to identify and enroll eligible participants for treatment, a process that was tested and refined during a census survey at the beginning of the project. Participants’ information is accessed from a cloud-based database (employing SurveyCTO software) via the smartphone, using either a fingerprint verification (employing Simprints technology) [13] or an identification (ID) card given to each individual during the baseline census survey. If no match is found for a fingerprint or no ID card is presented, the HEW searches the database using the name given by the participant. Anyone not registered during the census and/or previous rounds of MDA will be recorded and enrolled as a new participant during the current round of MDA when they are approached for treatment.

The treatment data for all the other districts are collected using an Excel sheet prepared by the FMoH for reporting purposes. The data are then entered into the District Health Information Software 2 (DHIS2) platform [4] on completion of the MDA. Upon completion of each MDA round, a coverage survey is conducted by the Ethiopian Public Health Institute ((EPHI) in selected districts according to the design recommended by WHO [18], to verify the MDA coverage reported by the FMoH. It should be noted that the method recommended by WHO for this type of survey is subject to recall bias.

Between 2015 and 2017, all districts in the Geshiyaro project received sMDA for STH annually. Starting in 2019, with the initiation of the Geshiyaro project, MDA expanded to cover adults in Arms 1 and 2, whereas districts in Arm 3 have continued with sMDA targeting SAC. Since the beginning of 2021, children between the ages of 12 and 23 months and pre-SAC in Arms 1 and 2 have been included in STH mass treatment using ALB syrup or tablets, respectively.

The treatment was given in Bolosso Sore or Arm 1 pilot district for five rounds using ALB before the fourth annual follow-up survey (FU4) in 2022. The other districts in Arm 1 and Arm 2 received three rounds of MDA before the third annual follow-up survey (FU3) in 2023. Districts in Arm 3 received only one round of treatment before the second follow-up survey (FU2) in 2023 due to the interruption of MDA by the COVID pandemic, resource limitations, and national schistosomiasis (SCH)/STH remapping activities (Table 1). In light of the varying intervention implementation duration in the different arms, we subdivide Arm 1 into the “Arm 1 pilot,” which consists of the pilot Bolosso Sore district, and “Arm 1” as the remaining four districts (Bolosso Bombe, Damot Gale, Damot Pulasa, Damot) in which implementation of control activities were initiated 1 year after the 2018/2019 pilot (see Table 1).

**Table 1:** MDA implementation by year and arm in the Geshiyaro project

Arm	Districts	Year	Round 1 (R1)	Round 2 (R2)	Drug	MDA treatment record
Arm 1 pilot Bolosso Sore (BS)	Year 1 (2018/2019)	Jan. 2019	NA	ALB	Smartphone (biometric, ID card, and name search)	
	Year 2 (2019/2020)	Nov. 2019	NA	ALB		
	Year 3 (2020/2021)	Nov 2020	NA	ALB		
	Year 4 (2021/2022)	Dec. 2021	May 2022	ALB		
Arm 1	Bolosso Bombe, Damot Gale, Damot Pulasa, Damot Sore (four districts in Arm 1-FD)	Year 1 (2018/2019)	NA	NA		Program report (paper-based)
		Year 2 (2019/2020)	NA	NA		
		Year 3 (2020/2021)	Nov. 2020	NA	ALB	
		Year 4 (2021/2022)	Dec. 2021	May 2022	ALB	
Arm 2	Damot Weydie Abala Abeya	Year 1 (2018/2019)	NA	NA		Smartphone (biometric, ID card and name search)
		Year 2 (2019/2020)	NA	NA		
		Year 3 (2020/2021)	Nov 2020	NA	ALB	
		Year 4 (2021/2022)	Dec. 2021	May 2022	ALB	
Arm 2	Araka town, Bayra Koisha, Boditi town, Dugna Fango, Gesuba town, Genuno town, Hobicha, Humbo, Kawo Koisha, Kindo Didaye, Kindo Koisha, Ofa, Sodo town, Sodo Zuria	Year 1 (2018/2019)	NA	NA		Program report (paper-based)
		Year 2 (2019/2020)	NA	NA		
		Year 3 (2020/2021)	Nov. 2020	NA	ALB	
		Year 4 (2021/2022)	Dec. 2021	May 2022	ALB	
Arm 3	Wondo, Wondo Genet, Hula	Year 4 (2021/2022)	Dec. 2021	NA	ALB	Program report (paper-based)

The WaSH intervention, which was comprehensive in Arm 1 led by World Vision Ethiopia (WVE), aims to increase safe water coverage to 85% and basic sanitation coverage to 82% by the end of the project. In addition, it includes behavioral communications to address local knowledge gaps related to handwashing, shoe-wearing, and toilet utilization. The set targets are yet to be achieved, and progress plus impact will be covered in a separate publication.

### Longitudinal parasitological sentinel surveys

A baseline parasitological mapping survey was conducted in all arms in 2018 to determine the prevalence and intensity of STH infection in the identified communities, stratified by age and gender. The baseline data showed low levels of schistosome parasite (both *Schistosoma mansoni* and *Schistosoma haematobium*) infection in all study sites using the Kato–Katz (KK) diagnostic method [15]. The focus of this paper will be on the control of STH infection. Data for the schistosome infections has been published elsewhere [19].

Based on the results from the baseline mapping survey stratified by infection intensity as either low, moderate, or high STH infection [20], 45 sites (15 from each arm) and 150 participants from each site were selected for longitudinal parasitological surveys, conducted before each annual round of MDA (Table 2 provides more information on the timeline of baseline and follow surveys). The baseline data has been published separately [21].

**Table 2:** Timing of the longitudinal parasitological surveys conducted in the Geshiyaro project sentinel sites over the first 5 years of the project

Arm	Baseline	Follow-up survey 1 (FU1)	Follow-up survey 2 (FU2)	Follow-up survey 3 (FU3)	Follow-up survey 4 (FU4)
Arm 1 pilot	Nov. 2018	Oct. 2019	Oct. 2020	Nov. 2021	Nov. 2022
Arm 2 (FD)	Oct. 2020	Nov. 2021	Apr. 2022	Apr. 2023	
Arm 3	Oct. 2020	Nov. 2021	Apr. 2022	Apr. 2023	
Arm 4	Oct. 2020	Apr. 2022	Apr. 2023		

Participants included in the longitudinal parasitological survey were tested for STH infection each year before the MDA employing KK methods using two-day stools (two slides from each and thus four in total over the 2 days) collected from the participants. Participants in the longitudinal cohort were asked to provide stools in labeled cups for two consecutive days. Four thick smears from the two consecutive days of stool samples were prepared. The stool was tested for the presence of STH eggs or larvae using the KK diagnostic method, and eggs were counted to determine the intensity of infection [21]. The test result was read by trained laboratory technologists employing a light microscope. The number of eggs per gram (epg) of stool was calculated and categorized according to the WHO intensity classification (Additional file 4: Table S4) [22]. Participants’ biometric fingerprints and test results were recorded and linked to demographic and treatment data collected via smartphones. The data were stored using SurveyCTO software (Dobility, Inc., Cambridge, MA, USA). Full details of the procedures adopted are published in the Geshiyaro protocol paper [13].

### Statistical analysis

Descriptive epidemiological statistics were used to determine the two key parasitological indicators: the prevalence and the mean intensity of infection (measured by KK egg counts). Data analysis was performed in RStudio software. The changes in prevalence and intensity of infection within arms were compared using a *t*-test, with a log transformation of raw egg counts due to the underlying negative binomial distribution of egg counts per person. A generalized linear model was used to calculate the test of significance and make comparisons between the different arms. These comparisons are problematic due to the impact of the COVID-19 pandemic in influencing the timing and duration of control interventions.

Chi-square tests for independence were used to assess relationships between infection prevalence and gender or infection prevalence and age. An analysis of variance (ANOVA) was performed to assess whether there were significant differences in infection intensity (mean egg count over four slides) between the different age groups and two genders. The relative reduction in prevalence was calculated using the formula [prevalence at baseline − prevalence at follow-up]/[prevalence at baseline]. A bootstrap re-sampling method was used to calculate the confidence limits in the relative reduction of prevalence. The difference in differences (DID) was calculated using the formula [follow-up prevalence of intervention − baseline prevalence of intervention] − [follow-up prevalence of control − baseline prevalence of control]. The prevalence of any STH at baseline was compared with follow-up surveys in all arms, but only moderate *A. lumbricoides* infection was used to compare the changes in the intensity of infection because there were no moderate or heavy infections for the other STH species. The prevalence of any STH was determined by considering the presence of a single egg from one of the three major species (*A. lumbricoides*, *T. Trichiura*, hookworm) during sample testing.

## Results

### Participants enrolled

A total of 568 (mean age = 24 years, standard deviation [SD] = 16.3) and 606 (mean age = 23.5 years, SD = 17.3) individuals participated in the Arm 1 pilot in the baseline and follow-up 4 (FU4) surveys, respectively (see Tables 1 and 2 for timing of MDA and sentinel site surveys by arm). In the four other districts in Arm 1, there were 1580 (mean age = 20.7 years, SD = 16.1) participants at the baseline and 1648 (mean age = 21.6 years, SD = 16.6) participants in the follow-up survey 3 (FU3). In Arm 2, a total of 2295 (mean age = 20.4, SD = 15.5) participated at baseline, and 2249 (mean age = 20.9 years, SD = 15.8) participated in follow-up survey 3 (FU3). Additionally, 2051 (mean age = 19.8 years, SD = 14.7) enrolled at baseline, and 2257 (mean age = 19.8, SD = 14.7) in follow-up survey 2 (FU2) in Arm 3 (Additional file 1: Table S1). The participants were identified at each longitudinal survey and linked to the information captured previously at baseline or at later surveys. If any participants were not found during a survey, they were replaced by individuals with similar demographic characteristics.

### Prevalence and intensity of STH infection at baseline

The overall prevalence of any STH infection at baseline in the Arm 1 pilot was 34.5% in 2018/2019. *Ascaris lumbricoides* was the dominant species (30.2%), followed by *T. trichiura* (9.3%) and hookworm (6.5%). The prevalence of any STH in Arm 1 was 27.4% (18.7% for *A. lumbricoides*, 8.7%, for hookworm, and 5.2% for *T. trichiura*) in 2020. In Arm 2, the prevalence of any STH was 23%. In contrast to the other arms, hookworm was the most prevalent of all the STH species (14.8% for hookworm, 8.8% for *A. lumbricoides,* and 1.6% for *T. trichiura*) in 2020. Nearly half (49.6%) of the participants in Arm 3 were infected with one of the STH species (43.8% for *A. lumbricoides*, 13.2% for hookworm, and 10.6% for *T. trichiura*) in 2020.

Among the study arms, the highest mean epg for *A. lumbricoides* was recorded in Arm 3, reaching 857.2 (95% confidence interval [CI] 725.9–999.8). The next highest was in the Arm 1 pilot, which had a mean epg of 457.5 (95% CI 302.8, 687.2), followed by Arm 1 with a mean epg of 329.5 (95% CI 244, 428), and finally, Arm 2 with a mean epg of 145.2 (95% CI 91, 212). Most of the infections for the three species were light, with no instances of heavy *A. lumbricoides* or *T. trichiura* infection (Table 3).

**Table 3:** Baseline prevalence and mean intensity of STH infection in longitudinal sentinel site surveys by arm (95% confidence limits in brackets for the positive binomial distribution of prevalence values and negative binomial distribution of intensity of infection measures)

	Arm 1 pilot	Arm 1	Arm 2	Arm 3
Total any STH infection (% and 95% CI)	34.5 (30.6, 38.5)	27.4 (25.2, 29.7)	23 (21.3, 24.8)	49.6 (47.4, 51.7)
*A. lumbricoides* (% and 95% CI)	30.2 (26.5, 34.2)	18.7 (16.6, 20.8)	8.8 (7.7, 10.1)	43.8 (41.7, 45.9)
Hookworm (% and 95% CI)	6.5 (4.6–8.9)	8.7 (7.4, 10.3)	14.8 (13.4, 16.4)	13.2 (11.9, 14.8)
*T. trichiura* (% and 95% CI)	9.3 (7.1, 12.1)	5.2 (6.1, 6.4)	1.6 (1.1, 2.2)	10.6 (9.4, 12)
*A. lumbricoides* (mean epg and 95% CI)	457.5 (302.8, 687.2)	329.5 (244, 428)	145.2 (91, 212)	857.2 (725.9–999.8)
Hookworm (mean epg and 95% CI)	4.3 (1.7, 8.2)	8 (5, 11.7)	32.7 (22.4, 42.4)	11.5 (8.6, 14.9)
*T. trichiura* (mean epg and 95% CI)	12.6 (5.6, 22.7)	19.3 (9.3, 32.8)	16.1 (8.2, 26.8)	11.2 (7.9, 15)
*A. lumbricoides* infection level category				
Heavy (%)	0	0	0	0
Moderate (%)	2.6 (1.5, 4.4)	1.5 (0.9, 2.2)	0.4 (0.2, 0.8)	4 (3.2, 4.9)
Light (%)	27.6 (24, 31.4)	17.2 (15.4, 19.2)	8.4 (7.3, 9.6)	39.8 (37.8, 41.9)
Hookworm infection category				
Heavy (%)	0	0	0.01 (0.03, 0.4)	0
Moderate (%)	0	0	0.04 (0.02, 0.3)	0.05 (0.02, 0.3
Light (%)	6.5 (4.7, 8.9)	8.7 (7.4, 10.3)	14.7 (13.3, 16.2)	13.2 (11.8, 14.7)
*T. trichiura* infection category				
Heavy (%)	0	0	0	0
Moderate (%)	0.2 (0.009, 1.1)	0.6 (0.3, 1.2)	0.6 (0.4, 1.1)	0.1 (0.04, 0.4)
Light (%)	9.1 (6, 11.9)	4.6 (3.4, 5.8)	0.9 (0.6, 1.5)	10.5 (9.2, 11.9)

### Prevalence and intensity of STH infection in the follow-up surveys

The overall prevalence of any STH infection in the Arm 1 pilot was 10.6 (95% CI 8.3, 13.4), while *A. lumbricoides* remained the most common infection during the FU4 survey in 2022 (6.8%, 3%, and 3.2% for *A. lumbricoides*, hookworm, and *T. trichiura*, respectively). The prevalence of any STH infection was found to be 5.5% (95% CI 5 4.4, 6.7) in Arm 1 and 4.5% (95% CI 3.7, 5.5) in Arm 2 during the FU3 survey in 2022. Hookworm infection in Arm 2 decreased and became the second most common infection during the follow-up survey, despite being the dominant species at the baseline, whereas the prevalence of any STH in Arm 3 remained high (26.1%, 95% CI 24.2, 27.9) during the FU2 survey in 2023 (Table 4).

**Table 4:** Prevalence and intensity of STH infection in the follow-up longitudinal sentinel site surveys (95% confidence limits in brackets)

	Arm 1 pilot	Arm 1	Arm 2	Arm 3
Total any STH infection (% and 95% CI)	10.6 (8.3, 13.4)	5.5 (4.4, 6.7)	4.5 (3.7, 5.5)	26.1 (24.2, 27.9)
*A. lumbricoides* (% and 95% CI)	6.8 (5, 9.1)	4.6 (3.7, 5.8)	2.9 (2.3, 3.7)	23.3 (21.6, 25.1)
Hookworm (% and 95% CI)	3 (1.8, 4.7)	0.9 (0.5, 1.5)	1.3 (0.9, 1.9)	2.2 (1.7, 2.9)
*T. trichiura* (% and 95% CI)	3.2 (2.1, 5.1)	0.5 (0.2, 1)	0.8 (0.5, 1.2)	7.4 (6.3, 8.5)
*A. lumbricoides* (mean epg and 95% CI)	33.9 (0.8, 69.2)	78.5 (53.7, 108)	52.8 (23.2, 94.3)	520.6 (402.1, 654.1)
Hookworm (mean epg and 95% CI)	1.4 (0.3, 3.3)	0.2 (0.1, 0.4)	0.2 (0.1, 0.4)	3.5 (1.4, 6.4)
*T. trichiura* (mean epg and 95% CI)	0.8 (0.2, 1.7)	2.7 (0.1, 9.4)	2.7 (0.2, 8.4)	5.9 (3.9, 8.2)
*A. lumbricoides* infection category				
Heavy (%)	0	0	0	0
Moderate (%)	0	0.5 (0.3, 1.1)	0.08 (0.01, 0.4)	2.8 (2.2, 3.6)
Light (%)	6.6 (4.8, 9)	4.1 (3.2, 5.2)	2.8 (2.2, 3.6)	20.5 (18.8, 22.2)
Hookworm infection category				
Heavy (%)	0	0	0	0
Moderate (%)	0	0	0	0
Light (%)	3 (1.8, 4.7)	0.9 (0.5, 1.5)	1.3 (0.9, 1.9)	2.2 (1.6, 2.9)
*T. trichiura* infection category				
Heavy (%)	0	0	0	0
Moderate (%)	0	0.06 (0.003, 0.4)	0.09 (0.02, 0.4)	0.04 (0.002, 0.3)
Light (%)	3.3 (2.1, 5.1)	0.4 (0.2, 0.9)	0.7 (0.4, 1.1)	7.3 (6.3, 8.5)

Arm 3 was found to have the highest *Ascaris* mean epg (520.6, 95% CI 402.1, 654.1), followed by Arm 1 (78.5, 95% CI 53.7, 108), Arm 2 (52.8, 95% CI 23.2, 94.3), and finally, the Arm 1 pilot (33.9, 95% CI 0.8, 69.2) (Table 4).

In the follow-up surveys, the majority of infections remained light, with no heavy infection for *A. lumbricoides* or *T. trichiura*, and no heavy or moderate infection for hookworm (Table 4).

### Frequency distribution of *A. lumbricoides* eggs per gram of feces

**Fig. 2:**
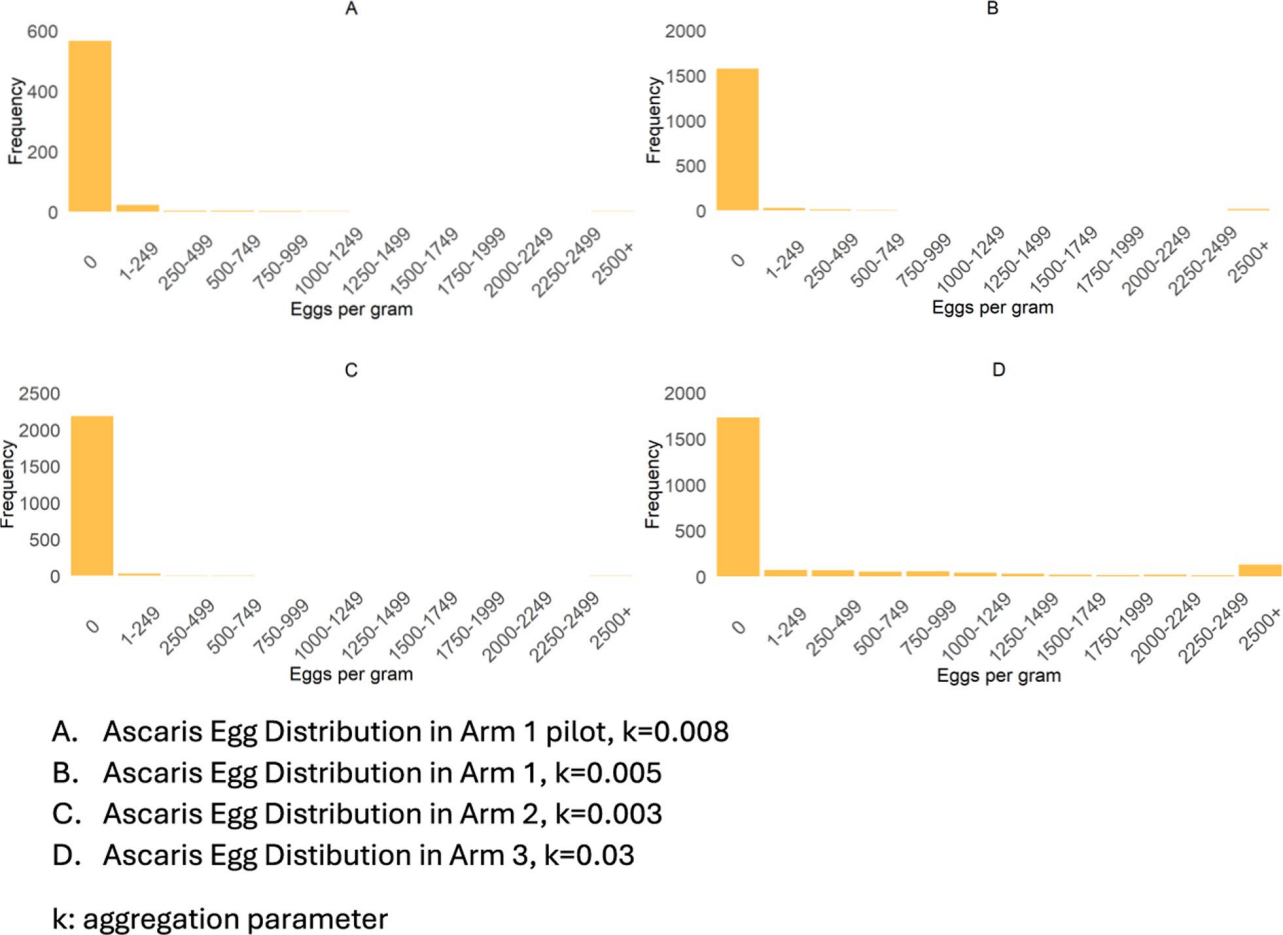
Frequency distribution of *Ascaris lumbricoides* egg count in **A** Arm 1 pilot, **B** Arm 1, **C** Arm 2, **D** Arm 3

Figure 2 shows the frequency distributions of the intensity of infection, measured as epg of feces, across Arms 1 to 3. The negative binomial distribution was fitted to these observed distributions and the *k* values were calculated by a maximum likelihood method [23]. The parameter *k* of the negative binomial distribution is an inverse measure of the degree of parasite aggregation in the human host population [24]. The *k* values are recorded in each graph. They are very low, indicating very high aggregation of worms per person, as reflected indirectly by the egg counts. The egg intensity of infection measure was highest in Arm 3, where only sMDA was implemented, versus other arms implementing cMDA. The results from the baseline and follow-up surveys are shown in Additional file 1: Table S2 and Table S3 show, which indicate that the overall *A. lumbricoides* mean egg count showed a decreasing pattern over the years of treatment, and this trend was comparable across all age groups in each arm.

Regarding hookworm and *T. trichiura,* the mean egg count remained very low in all age groups, making comparisons across time periods challenging given the low numbers infected.

### Effect of interventions on the prevalence and intensity of STH infection at different sampling times in the sentinel sites

The overall STH prevalence was significantly reduced from the baseline findings in all arms in the follow-up surveys. In the Arm 1 pilot, it decreased from 34.5% (30.6%, 38.5%) at baseline to 10.6% (8.3%, 13.4%) (*df* = 1, *P* < 0.0001) in FU4. In Arm 1, it dropped from 27.4% (25.2%, 29.7%) at baseline to 5.5% (4.4%, 6.7%) in FU3 (*df* = 1, *P* < 0.0001). Arm 2 showed a reduction from 23% (21.3%, 24.8%) at baseline to 4.5% (3.7%, 5.5%) in FU3 (*df* = 1, *P* < 0.0001). In Arm 3, the prevalence decreased from 49.6% (47.4%, 51.7%) to 26.1% (24.2%, 27.9%) in FU2 (*df* = 1, *P* < 0.0001).

**Fig. 3:**
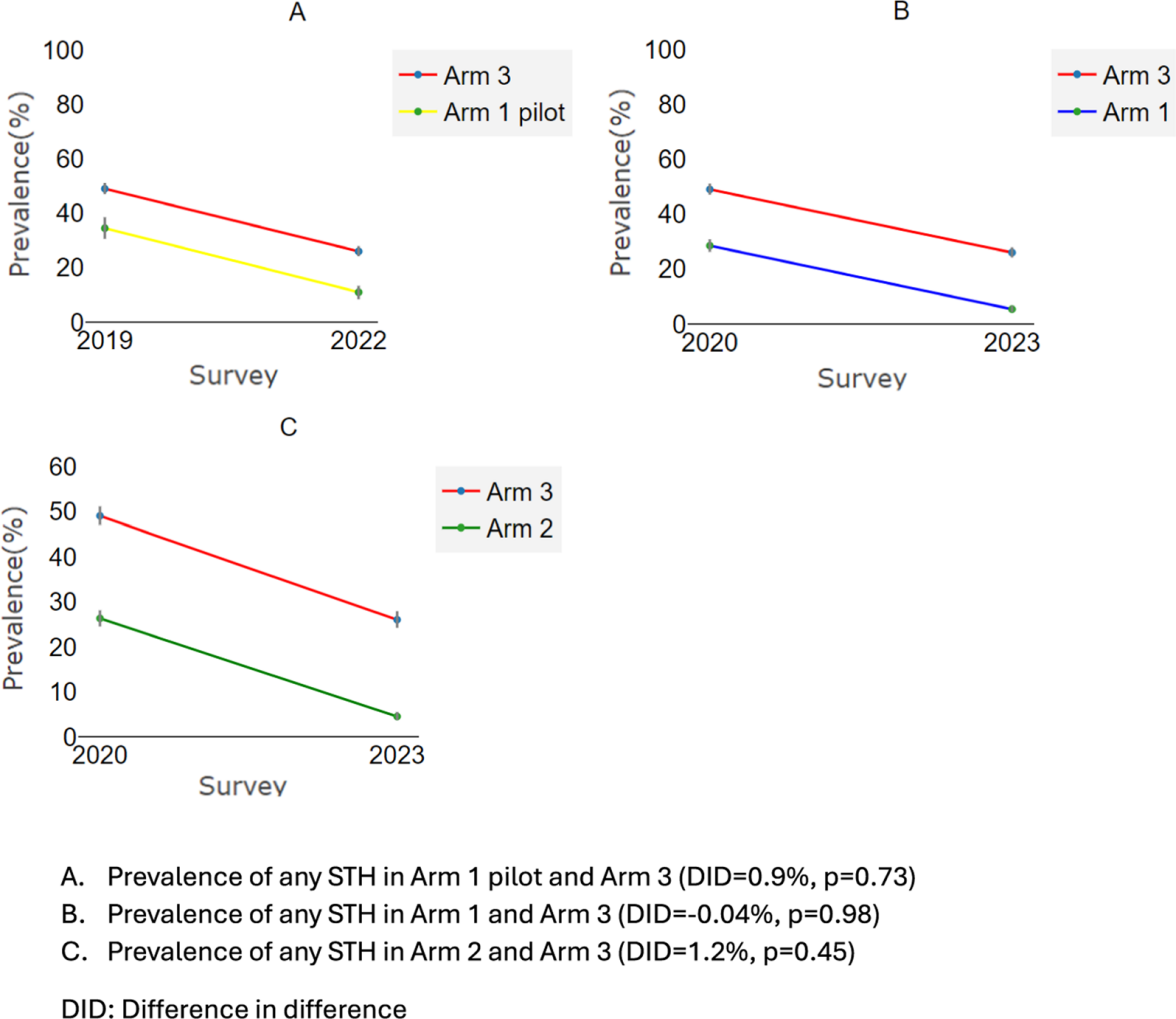
Reduction in any STH infection using relative difference measures (DID) to account for different starting prevalence at baseline in the different arms. **A** Reduction from Arm 1 pilot to Arm 3; **B** reduction from Arm 1 to Arm 3; **C** reduction from Arm 2 to Arm 3. DID: difference in difference

The relative reduction in the prevalence of any STH was the highest in Arm 2, with a decrease of 82.5% (79.3%, 84.2%), followed by Arm 1 with a reduction of 80.1% (75.3%, 84.6%), then the Arm 1 pilot with a decrease of 69.4% (60.1%. 76.6%), and lastly Arm 3 with a reduction of 46.9% (43.6%, 51%) (Fig. 3).

**Fig. 4:**
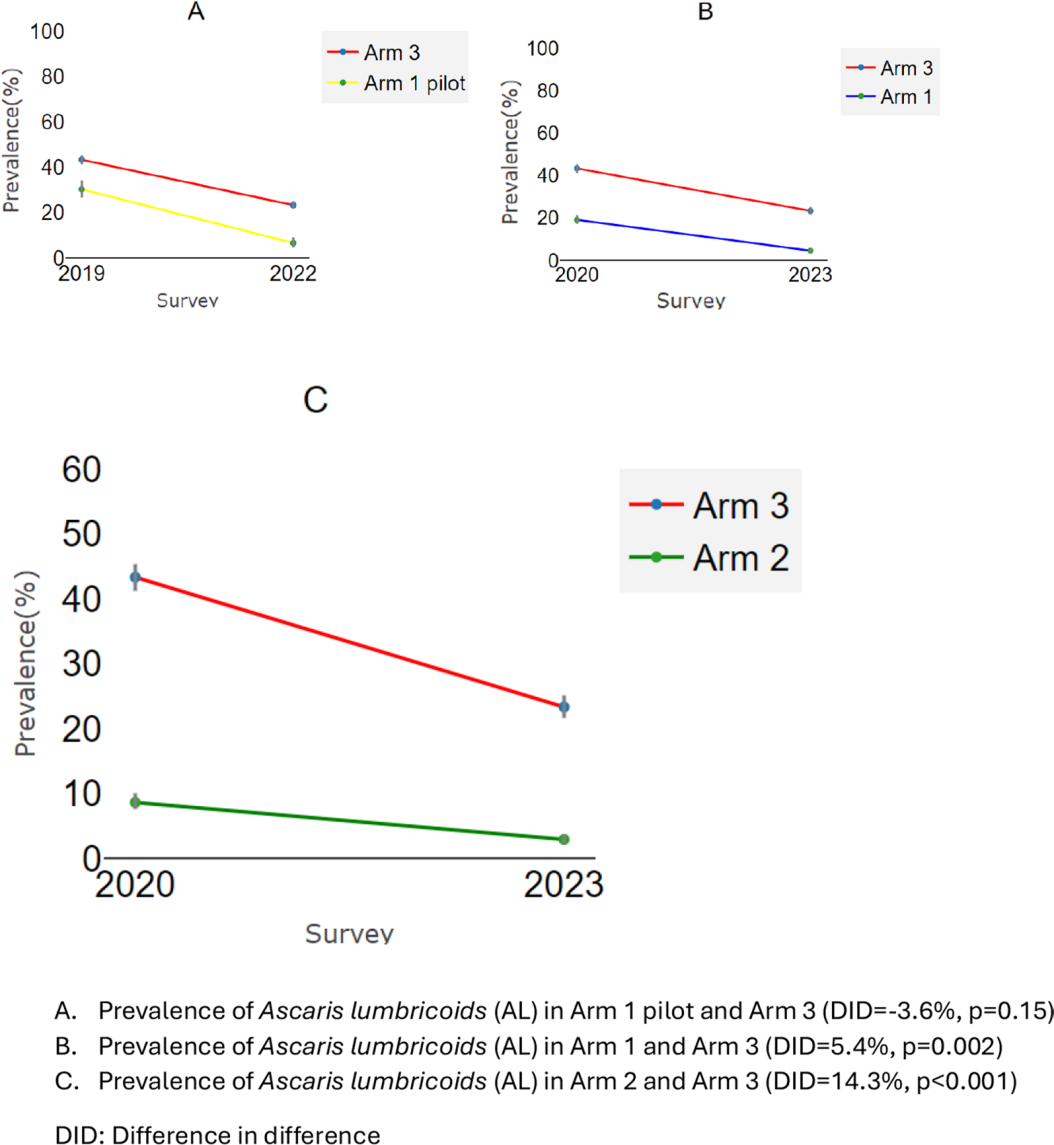
Reduction in *A. lumbricoides* infection using relative difference measures (DID) to account for different starting prevalence at baseline in the different arms. **A** Reduction in Arm 1 pilot compared to Arm 3; **B** reduction in Arm 1 compared to Arm 3; **C** reduction in Arm 2 compared to Arm 3. DID: difference in difference

The prevalence of *A. lumbricoides* infection, the dominant STH species in the study area, decreased from 30.2% (26.5%, 34.2%) to 6.8% (5%, 9.1%) in the Arm 1 pilot, from 18.7% (16.6%, 20.8%) to 4.6% (3.7%, 5.8%) in Arm 1, from 8.8% (7.7%, 10.1%) to 2.9% (2.3%, 3.7%) in Arm 2, and from 43.8% (41.7%, 45.9%) to 23.3% (21.6%, 25.1%) in Arm 3, representing a reduction of 77.5% (70.8–84.7%) in the Arm 1 pilot, 75.4% (69.7–81.4%) in Arm 1, 67% (55.5–74.7%) in Arm 2, and 46.8% (41.2–50.6%) in Arm 3. The DID between the Arm 1 pilot and Arm 3 was −3.6% (*df* = 5685, *P* = 0.15), between Arm 1 and Arm 3 was 5.4% (*df* = 7733, *P* = 0.002), and between Arm 2 and Arm 3 was 14.3 (*df* = 9048, *P* < 0.001) (Fig. 4).

**Fig. 5:**
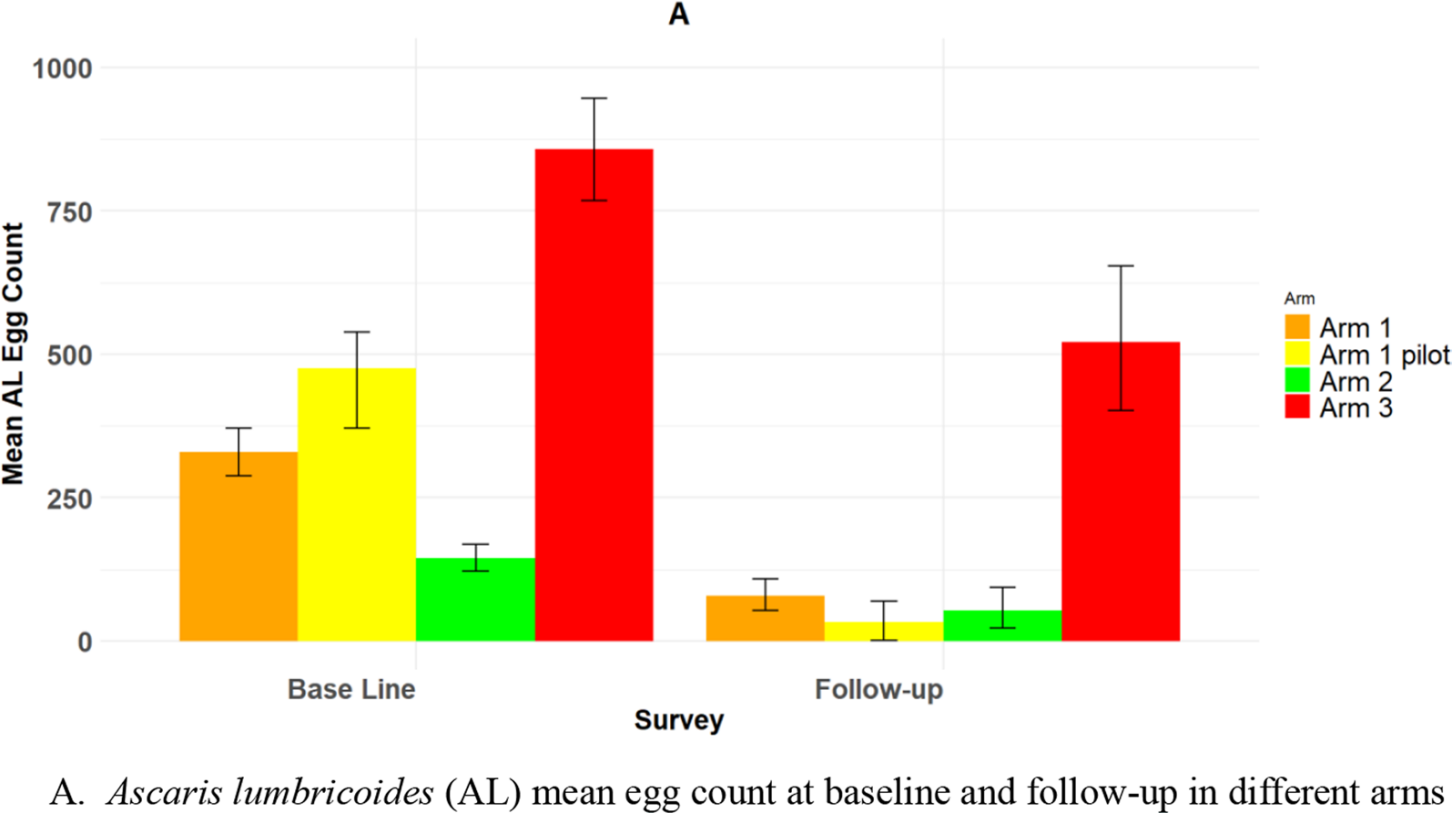
Mean *A. lumbricoides* (AL) egg count at baseline and follow-up survey in the different arms of the study

The overall mean *A. lumbricoides* egg reduction between different arms at baseline and follow-up surveys is shown in Fig. 5. In the follow-up surveys, the mean intensity of *A. lumbricoides* infection, as measured by epg, significantly decreased in all arms using the paired *t*-test on log-transformed data.

The prevalence of STH and *A. lumbricoides* was higher in female hosts across all arms except Arm 2, although the observed difference was not always statistically significant. The mean *A. lumbricoides* egg count was higher in males in Arm 2 (*df* = 1353, *P* = 0.02), while it was significantly higher in females in Arm 3 (*df* = 2021, *P* = 0.03).

**Fig. 6:**
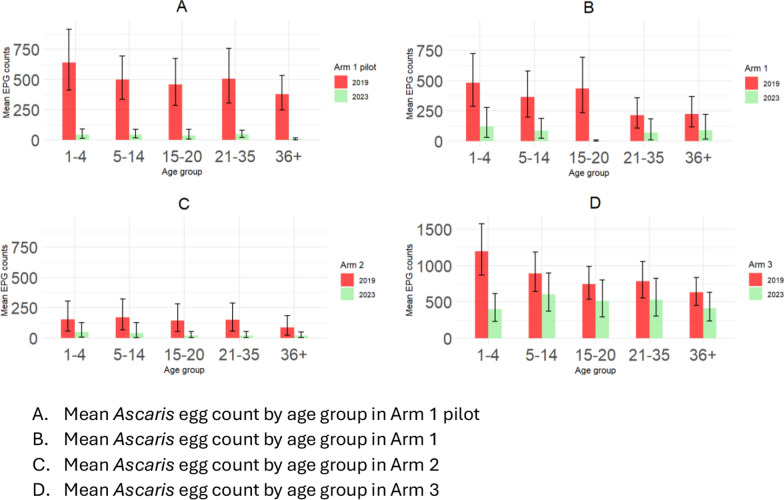
Patterns of the mean intensity of *A. lumbricoides* infection (mean egg counts) by age group and arm at baseline and follow-up surveys displayed as bar plots. **A** Arm 1 pilot; **B** Arm 1; **C** Arm 2; **D** Arm 3

STH and *A. lumbricoides* infection prevalence was reduced across all arms and age groups. However, the mean egg count was relatively higher in the age groups of 1–4 and 5–14 years. In the Arm 1 pilot, the mean egg count was reduced across all age groups. In Arm 1, reduction was observed in the age groups of 1–4, 5–14, and 15–20 years. Arm 2, which has a lower egg count than the other arms, showed a reduction in the age groups of 15–20 and 20–35 years, while in Arm 3, the mean egg count was higher at baseline and follow-up than in all other arms, with no significant reduction (*df* = 55.5, *P* = 0.15) except in the age group of 1–4 years, as shown in Fig. 6.

## Discussion

This paper examines the effect of combining MDA, WaSH, and BCC programs on the prevalence and mean intensity of STH infection in three different arms of the Geshiyaro study at a midpoint in the progress of the control project. Each arm implemented different control measures but for varying periods due to the interruptions in planned activity because of the COVID-19 pandemic.

*Ascaris lumbricoides* was the dominant STH species detected during the baseline and the follow-up surveys in the longitudinal sentinel sites, followed by hookworm and *T. trichiura*. Both hookworm and *T. trichiura* were at low prevalence levels in the study locations within Arms 1 and 3. An exception to this pattern was recorded in Arm 2, where hookworm was the commonest infection during the baseline survey. This pattern is in agreement with the national STH mapping conducted in Ethiopia between 2013 and 2014, where *A. lumbricoides* was found to be the most prevalent STH [5]. However, some published studies in Northern and Southern Ethiopia have identified hookworm as the dominant infection in specific sites, with *T. trichiura* being most common in southwestern Ethiopia. These variations are probably related to the difference in climatic conditions such as temperature and humidity that influence the survival of infective larvae and helminth eggs [25–27].

The prevalence of any STH infection decreased significantly in all study arms, with relative reduction of 69.4%, 80.1%, 82.2%, and 46.9% in the Arm 1 pilot, Arm 1, Arm 2, and Arm 3, respectively. Despite the implementation of intensive WaSH interventions in Arm 1, the relative reduction in Arm 2 was higher than in Arm 1. Possible contributing factors include the selection of districts for the different arms, which was based in part on their WaSH coverage before the initiation of the Geshiyaro project. This might have resulted in assigning districts with better WaSH coverage to Arm 2. Other factors include districts in Arm 1, particularly Bolosso Sore, which struggled to reach good MDA coverage targets during years 1 and 2 of MDA implementation. This was due to low acceptance of MDA, in part a result of some hesitation in the use of fingerprints as a method for verification of treatment. It may also be due in part to the fact that the effect of improved WaSH on STH transmission could take several years to become apparent [28, 29]. The relative reduction of any STH infection in Arm 3 was the smallest, potentially attributable to fewer MDA rounds and MDA being limited to SAC, leaving reservoirs of infection in adults to ensure continued transmission.

The majority of infections in the three arms were of light intensity at the baseline and in the follow-up surveys. Heavy infections were rare during both the baseline and the follow-up surveys. In the follow-up surveys, the prevalence of moderate *A. lumbricoides*, hookworm, and *T. trichiura* infections was less than 2%, but the prevalence of moderate *A. lumbricoides* infection in Arm 3 (2.8%) was above the WHO threshold (< 2%) for elimination as a public health problem (EPHP) [1].

The mean intensity of infection for *A, lumbricoides* was highest in the age groups of 1–4 and 5–14 years. This is in line with the commonly observed pattern of high infection in pres-SAC, typically reaching a peak in SAC before declining in adults [30].

The mean intensity of infection for *A. lumbricoides* remained low in all study arms. However, the mean intensity in Arm 3 remained significantly higher than that in the other arms. The reduction observed in all age groups in Arm 3 was less significant except for the 1–4-year age group. The higher intensity of infection in Arm 3 is probably attributable to the fewer rounds of MDA delivered and the focus on treating only SAC.

The distribution of epg followed a negative binomial distribution with small k-values, recording high degrees of parasite aggregation in the human host population in all the arms. As is typical for helminth infections in humans [31], the distribution is highly over-dispersed, with most harboring one or a few worms while a few harbor many. Worm presence is reflected in this study indirectly by epg of feces. The aggregation of eggs and hence adult parasites in fewer and fewer people as transmission declines due largely to MDA impact in this study poses some practical challenges for transmission elimination relating to how best to identify these few heavily infected individuals in communities who may be predisposed to repeated infection, thereby maintaining parasite transmission. Predisposition to repeated reinfection is likely to be caused by a combination of a number of factors including behavioral, social, genetic, and environmental variables [32]. A separate analysis of parasite distribution data from the Geshiyaro project has shown that a tendency of predisposition to high or low infection rates can confuse interpretation of the impact of treatment when the impact on infection sometimes appears unrelated to treatment history [33].

The observed benefit of cMDA in the Geshiyaro study aligns with recently reported findings in a number of studies that cMDA is better than sMDA treatment programs in reducing both the prevalence and intensity of infection [34, 35].

The major limitation in the interpretation of the findings from this study is that the interventions had different durations due to the disruptions in healthcare delivery arising from the COVID-19 pandemic. The pilot implementation was prolonged due to additional time needed in baseline mapping followed by baseline sentinel surveys. Resistance to biometric fingerprinting in the communities was also a factor in low initial MDA uptake. Bolosso Sore, the pilot district in the Arm 1 pilot, had 4 years of intervention compared to 3 years in Arms 1 and 2 and 2 years in Arm 3 before the midpoint follow-up surveys. Further delays occurred for Arm 3 due to national remapping of SCH infection and changes to STH treatment policy from school-based to include women of reproductive age. Hence, the findings from this midpoint analysis should be interpreted cautiously, in particular the apparent lack of impact of enhanced WaSH in Arm 1 when compared with Arm 2 results.

## Conclusions

Control interventions including MDA delivered to the whole community and not just SAC, combined with varying degrees of WaSH improvement, generated a significant reduction in the prevalence and mean intensity of STH infection within the Geshiyaro study communities in Southern Ethiopia up to the midpoint of the project. So far, cMDA has had the greatest impact on infection levels. Progress towards interrupting the transmission of STH in the Geshiyaro project communities is encouraging, but further efforts are needed to increase MDA coverage and compliance and enhance WaSH to achieve the project goal of < 2% prevalence.

### Supplementary Information

**Additional file 1: Table S1**. Participants enrolled, mean age in the longitudinal parasitological survey in Arms 1, 2, and 3.

**Additional file 2: Table S2**. Baseline mean intensity of infection (egg count) by age group in the longitudinal survey sites by STH species and arm.

**Additional file 3: Table S3**. Follow-up mean intensity of infection (egg count) in the longitudinal survey sites by species, age group, and arm with 95% CIs.

**Additional file 4: Table S4.** WHO classification of STH infections. *epg* egg per gram.

## Data Availability

All data analyzed in this study are included in this published article.

## Abbreviations

BCC: Behavioral change communication
CI: Confidence interval
epg: Eggs per gram
KK: Kato–Katz
MDA: Mass drug administration
NTD: Neglected tropical disease
SAC: School-age children
SCH: Schistosomiasis
SNNPR: Southern Nations, Nationalities, and Peoples’ Region
STH: Soil-transmitted helminth
WaSH: Water, sanitation, and hygiene
WHO: World Health Organization
