# Supplementary material for: Soil-transmitted helminth (STH) infections in the Wolaita zone in Southern Ethiopia: mid-stage evaluation of the Geshiyaro project and progress towards the interruption of transmission
Source: Parasit Vectors. 2024 Aug 21;17:355. doi: 10.1186/s13071-024-06422-2 (PMC11340125; doi:10.1186/s13071-024-06422-2)
Supplement: Supplementary file 1 — Additional file 1: Table S1. Participants enrolled, mean age in the longitudinal parasitological survey in Arms 1, 2, and 3. [file 13071_2024_6422_MOESM1_ESM.docx]

**Additional file 1 Table S1:** Participants enrolled, mean age in the longitudinal parasitological survey in Arm 1, 2, and 3

|  | Baseline (2019) | Follow-up 1 (2020) | Follow-up 2 (2021) | Follow-up 3 (2022) | Follow-up 4 (2022/23) |
| --- | --- | --- | --- | --- | --- |
| Arm 1 pilot | 568 | 588 | 553 | 589 | 606 |
| Arm 1 |  | 1580 | 1590 | 1662 | 1648 |
| Arm 2 |  | 2295 | 2083 | 2189 | 2249 |
| Arm 3 |  |  | 2151 | 2235 | 2257 |
