# Supplementary material for: Soil-transmitted helminth (STH) infections in the Wolaita zone in Southern Ethiopia: mid-stage evaluation of the Geshiyaro project and progress towards the interruption of transmission
Source: Parasit Vectors. 2024 Aug 21;17:355. doi: 10.1186/s13071-024-06422-2 (PMC11340125; doi:10.1186/s13071-024-06422-2)
Supplement: Supplementary file 2 — Additional file 2: Table S2. Baseline mean intensity of infection (egg count) by age group in the longitudinal survey sites by STH species and arm. [file 13071_2024_6422_MOESM2_ESM.docx]

**Additional file 2: Table S2:** Baseline mean intensity of infection (egg count) by age group in the longitudinal survey sites by STH species and Arm

|  | Arm 1 pilot | Arm 1 | Arm 2 | Arm 3 |
| --- | --- | --- | --- | --- |
| *Ascaris Lumbricoides* |  |  |  |  |
| 1-4 years | 639.5(36.5,2080.6) | 427.6(192,757) | 159.8(42.9,351.9) | 1211.1 (822.5,1673.6) |
| 5-14 years | 498.9(234.1,862) | 367.2(164.5,649.1) | 175.5(64.5,340.6) | 874.7(611.9,1184.8) |
| 15-20 years | 459.6(53.4,1291.7) | 449(142,928.6) | 146.8(42,315.7) | 740.5(493,1039.2) |
| 21-35 years | 506.2(154.7,1062.7) | 209.6(76.9,408.2) | 154.6(49.6,317.9) | 798.5(538.9,1107.2) |
| 36+ years | 377.6(141.6,726.9) | 231.2(85.6,447.8) | 82.3(16.3,200.7) | 641.2(420.2,909.2) |
| Hook worm |  |  |  |  |
| 1-4 years | 4.4(0,20.5) | 4.9(1.2,11.3) | 15.5(7.4,26.7) | 16.3(7.6,28.3) |
| 5-14 years | 3.6(0.6,9.3) | 4.3(1.1,9.7) | 11(5,19.3) | 8.8(4.7,14.3) |
| 15-20 years | 2.6(0,15.7) | 10.4(2.3,24.2) | 35.8(17.8,59.8) | 12.4(5.7,21.8) |
| 21-35 years | 4.5(0.2,15.9) | 12.6(4.9,23.8) | 27.5(14.5,44.6) | 5.6(2.6,9.6) |
| 36+ years | 5.8(0.8,15.8) | 9.2(3.1,18.6) | 80.4(39.5,135.8) | 16.4(8,27.5) |
| Trichuris |  |  |  |  |
| 1-4 years | 28.4(0.1,132.5) | 7(0.9,19.4) | 9.9(1,28.9) | 10.7(4.6,16.3) |
| 5-14 years | 23.7(5.7,54.5) | 12.9(3.2,29.3) | 31.3(0,196) | 12.6(6.3,21.1) |
| 15-20 years | 1.1(0,6) | 60.9(2.8,206.2) | 16(1.7,45.7) | 5(2.3,8.8) |
| 21-35 years | 7.7(1.2,19.8) | 9.3(0.6,30) | 3.5(0.1,13.3) | 9.7(4,17.8) |
| 36+ years | 3.4(0.4,9.7) | 20.5(1.7,61.9) | 17.3(0.3,67.1) | 18.5(5.3,39.7) |
