# Supplementary material for: Soil-transmitted helminth (STH) infections in the Wolaita zone in Southern Ethiopia: mid-stage evaluation of the Geshiyaro project and progress towards the interruption of transmission
Source: Parasit Vectors. 2024 Aug 21;17:355. doi: 10.1186/s13071-024-06422-2 (PMC11340125; doi:10.1186/s13071-024-06422-2)
Supplement: Supplementary file 3 — Additional file 3: Table S3. Follow-up mean intensity of infection (egg count) in the longitudinal survey sites by species, age group, and arm with 95% CIs. [file 13071_2024_6422_MOESM3_ESM.docx]

**Additional file: Table S3:** Follow-up mean intensity of infection (egg count) in the longitudinal survey sites by species, age group and Arm with 95% CIs.

| Age group | Arm 1 pilot | Arm 1 | Arm 2 | Arm 3 |
| --- | --- | --- | --- | --- |
| *Ascaris lumbricoides* |  |  |  |  |
| 1-4 years | 42(11.4,195.2) | 121.5(3.5,439.4) | 50(0.5,203.3) | 400.2(117.8,852.2) |
| 5-14 years | 44.6(3.2,139.4) | 86.4(21.1,197) | 41.6(5,115.9) | 604(397.7,858.6) |
| 15-20 years | 35.3(0,250) | 3.2(0,17.8) | 19(0.6,68.5) | 516.5(242.3,892.8) |
| 21-35 years | 43.7(6.7,121.2) | 70.2(3.4,235.2) | 21.3(2.2,61.7) | 533.6(284.2,860.2) |
| 36+ years | 6.1(0.6,29.4) | 90.7(10.8,253.5) | 19.2(2.1,54.6) | 411.8(218.2,665.9) |
| Hookworm |  |  |  |  |
| 1-4 years | 2.3(0,11.5) | 1.3(0,4.9) | 0.3(0,0.9) | 7.2(1.1,19) |
| 5-14 years | 1.3(0.1,4) | 0.02(0,1) | 0.6(0.2,1.1) | 1.2(0.1,2.4) |
| 15-20 years | 1.9(0,8.4) | 0 | 0.6(0.1,1.3) | 3.4(0.7,8.4) |
| 21-35 years | 1.9(0,9.7) | 0 | 3.8(0,3.12.1) | 3.4(1.1,7.1) |
| 36+ years | 0.4(0,2.1) | 0.2(0,0.6) | 4.1(0.2,13.5) | 6.1(2.3,11.7) |
| *Trichuris trichura* |  |  |  |  |
| 1-4 years | 0.1(0,0.6) | 0.2(0,0.6) | 0.1(0,0.2) | 3.4(1.4,6.2) |
| 5-14 years | 0.9(0,2.8) | 08.1(0.2,29.5) | 0.3(0,0.6) | 9.8(5.7,14.9) |
| 15-20 years | 1.5(0,6.4) | 0.6(0.1,1.9) | 2.4(0.1,7.9) | 4.3(2.1,7.2) |
| 21-35 years | 1.1(0,5.4) | 0.2(0,0.8) | 013.8(0.4,50.1) | 3.8(2.3,5.7) |
| 36+ years | 1(0,3.8) | 0 | 7(0.3,24.1) | 3.7(1.8,6.3) |
