# Supplementary material for: Soil-transmitted helminth (STH) infections in the Wolaita zone in Southern Ethiopia: mid-stage evaluation of the Geshiyaro project and progress towards the interruption of transmission
Source: Parasit Vectors. 2024 Aug 21;17:355. doi: 10.1186/s13071-024-06422-2 (PMC11340125; doi:10.1186/s13071-024-06422-2)
Supplement: Supplementary file 4 — Additional file 4: Table S4. WHO classification of STH infections. epg egg per gram. [file 13071_2024_6422_MOESM4_ESM.docx]

**Additional file: Table S4:** WHO classification of STH infections

| STH species | Light intensity (epg) | Moderate intensity (epg) | Heavy intensity (epg) |
| --- | --- | --- | --- |
| *Ascaris lumbricoides* | 1-4999 | 5000-49999 | >50000 |
| *Trichuris trichiura* | 1-999 | 1000-9999 | >10000 |
| Hookworms | 1-1999 | 2000-3999 | >4000 |
